# Direct Electrophysiological Correlates of Body Ownership in Human Cerebral Cortex

**DOI:** 10.1093/cercor/bhy285

**Published:** 2018-11-14

**Authors:** Arvid Guterstam, Kelly L Collins, Jeneva A Cronin, Hugo Zeberg, Felix Darvas, Kurt E Weaver, Jeffrey G Ojemann, H Henrik Ehrsson

**Affiliations:** 1 Princeton Neuroscience Institute, Princeton University, New Jersey, USA; 2 Department of Neuroscience, Karolinska Institutet, Stockholm, Sweden; 3 Department of Neurological Surgery, University of Washington, Seattle, WA, USA; 4 Departments Biomedical Engineering and Radiology, University of Washington, Seattle, WA, USA; 5 Department of Radiology, University of Washington, Seattle, WA, USA; 6 Integrated Brain Imaging Center, UW Radiology, Seattle, WA, USA

**Keywords:** body perception, electrocorticography, functional magnetic resonance imaging, rubber hand illusion

## Abstract

Over the past decade, numerous neuroimaging studies based on hemodynamic markers of brain activity have examined the feeling of body ownership using perceptual body-illusions in humans. However, the direct electrophysiological correlates of body ownership at the cortical level remain unexplored. To address this, we studied the rubber hand illusion in 5 patients (3 males and 2 females) implanted with intracranial electrodes measuring cortical surface potentials. Increased high-γ (70–200 Hz) activity, an index of neuronal firing rate, in premotor and intraparietal cortices reflected the feeling of ownership. In both areas, high-γ increases were intimately coupled with the subjective illusion onset and sustained both during and in-between touches. However, intraparietal activity was modulated by tactile stimulation to a higher degree than the premotor cortex through effective connectivity with the hand-somatosensory cortex, which suggests different functional roles. These findings constitute the first intracranial electrophysiological characterization of the rubber hand illusion and extend our understanding of the dynamic mechanisms of body ownership.

## Introduction

How does the brain shape the experience that my hand belongs to me? Historically, this question has been approached by studying the brain pathology that underlies the loss of limb ownership in certain neurological patients (Vallar and Ronchi 2009; Feinberg et al. 2010), and, more recently, using perceptual illusions to manipulate the sense of ownership over artificial limbs in healthy individuals (Botvinick and Cohen 1998; Ehrsson et al. 2004). The most influential of these illusions is the rubber hand illusion (RHI), in which viewing a rubber hand that is synchronously touched with one’s own hidden hand causes the rubber hand to be attributed to one’s own body (Botvinick and Cohen 1998). Although human neuroimaging studies using hemodynamic proxies of brain activity, such as functional magnetic resonance imaging (fMRI) (Ehrsson et al. 2004; Brozzoli et al. 2012; Guterstam et al. 2013) and positron emission tomography (PET) (Tsakiris et al. 2007), have shown that the illusion is associated with activity in multisensory cortical regions, the direct electrophysiological correlates of these findings remain to be quantified. To this end, we examined the RHI using electrocorticography (ECoG), which measures cortical surface potentials via electrode arrays that are directly placed on the brain’s surface.

Previous neuroimaging studies have shown that the RHI is associated with increased hemodynamic responses in multisensory areas in the ventral (v) and dorsal (d) premotor cortices (PMC) and along the intraparietal sulcus (IPS) (Ehrsson et al. 2004, 2005; Brozzoli et al. 2012; Gentile et al. 2013; Limanowski and Blankenburg 2016), which is compatible with behavioral evidence that suggests that the illusion depends on the basic multisensory congruence principles (Botvinick and Cohen 1998; Tsakiris and Haggard 2005; Stein and Stanford 2008). Although these results have been informative in several ways, the limitations of neuroimaging methods that are based on hemodynamic markers have resulted in several remaining important issues. For example: Are the observed fMRI activations reflected in neuronal discharge, in contrast to subthreshold depolarization or inhibition? What frequency band rhythm best reflects ownership activity? What are the precise temporal profiles of ownership-related brain activity in the PMC and IPS around the illusion onset, and in relation to the applied tactile stimulation? And, how is input from the primary somatosensory cortex (SI) integrated into higher multisensory areas, where the visuotactile integration process that underlies the RHI supposedly occurs (Makin et al. 2008; Tsakiris 2010; Ehrsson 2012; Blanke et al. 2015)?

To address these questions, we examined the neural activity that is associated with the RHI using ECoG in 5 patients who were implanted with arrays of subdural electrodes for localizing medically intractable epilepsy in preparation for resective brain surgery. The recording of each 2.3-mm-diameter ECoG electrode (1 cm interelectrode distance) captures the electrophysiological signal from the underlying population of neurons with high temporal resolution, signal-to-noise ratio and anatomical accuracy (Ritaccio et al. 2014). Thus, this method was ideal for addressing the aims of this study. In addition, 2 participants completed an fMRI experiment prior to the electrode implantation, which allowed for a direct comparison of hemodynamic fMRI and electrophysiological ECoG responses. Finally, one participant underwent a sensory stimulation screening procedure that functionally localized the hand representation of SI with high precision, which permitted us to examine illusion-related changes in the flow of information from SI to higher-order sensory areas in the posterior parietal lobe to illustrate the mechanisms through which tactile input is integrated in multisensory areas to shape the feeling of limb ownership.

We primarily focused our ECoG analysis on changes in high-γ broadband (70–200 Hz) activity, which, unlike oscillatory activity in the α, β, or γ bands, represents a reliable electrophysiological index of average neuronal population firing (Miller et al. 2007; Ray and Maunsell 2011; Suffczynski et al. 2014). Given the evidence from previous fMRI studies on the RHI (Ehrsson et al. 2004, 2005; Tsakiris et al. 2007; Brozzoli et al. 2012; Gentile et al. 2013), we hypothesized that the experience of the illusion would be coupled with high-γ activity in the premotor and intraparietal cortices. We also predicted that ownership of the rubber hand would lead to a change in connectivity between SI and IPS, reflecting the integration process of tactile signals into a multisensory body representation within the IPS (Makin et al. 2008; Tsakiris 2010; Ehrsson 2012; Blanke et al. 2015).

**Figure 1.**
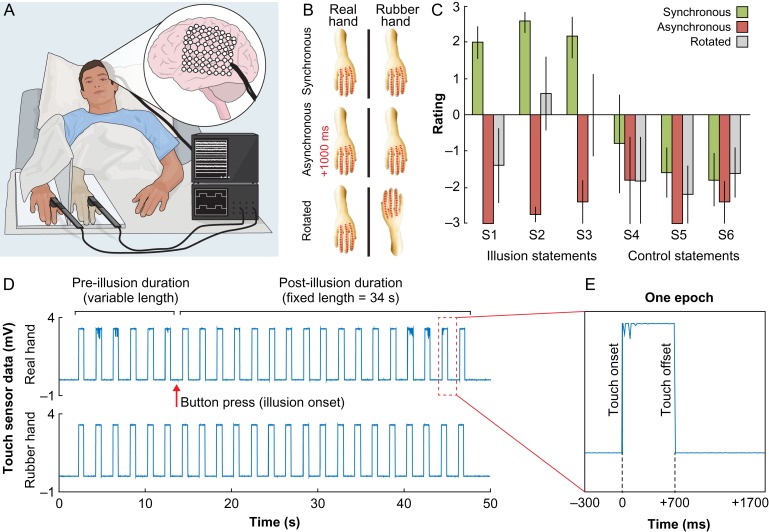
(*A*) Experimental setup. The illusion was elicited in 5 patients who were implanted with intracranial arrays of electrodes, through the synchronous stroking of a rubber hand and the unseen real hand, using 2 touch probes. (*B*) Experimental design. There were 3 experimental conditions: synchronous (illusion) or asynchronous stroking of the hands (control), or synchronous stroking using a rotated rubber hand (control). The red dotted lines indicate the fingers that were stroked. (*C*) Behavioral results. The synchronous condition was associated with significantly higher ratings on the questionnaire statements (Table 1) that were designed to capture the illusion experience (S1–S3) compared with the asynchronous and rotated control conditions. There was no difference for the control statements (S4–S6). Errors bars denote the SEM. (*D*) Illusion elicitation. The experimenter stroked both hands in synchrony until the participant pressed a button to indicate the illusion onset, after which the experimenter continued to stroke for 34 s. (*E*) Data segmentation. For the main analyses (Fig. 2*A*), the ECoG data were segmented into 2000-ms epochs that were aligned with the onset of tactile stimulation.

To test our hypotheses, we exposed participants to the RHI by synchronously stroking a rubber hand and their real hand, which was occluded from view (Fig. 1*A*), and 2 established control conditions that consisted of asynchronous stroking or rotating the rubber hand through 180° (Fig. 1*B*) (Botvinick and Cohen 1998; Ehrsson et al. 2004). Here, we report a set of neuronal populations in the premotor and intraparietal cortices that consistently show increased high-γ activity—that overlap with blood-oxygen-level dependent (BOLD) responses—during periods of ownership of the rubber hand and that the temporal profiles of the increase in activity mirror the subjective illusion onset. Interestingly, the illusion-related high-γ activity in the IPS and PMC was sustained during as well as in-between individual touches; however, the activity in IPS was modulated by the applied tactile stimulation to a significantly higher degree than the PMC, which suggests potentially distinct functional roles of these 2 key areas in generating the RHI. Finally, we demonstrate that activity in the hand-SI cortex significantly predicts IPS activity 200 ms later in an illusion-specific manner, which reveals a neural mechanism for how low-level somatosensory signals are integrated into higher-order body representations in the posterior parietal lobe to shape the feeling of limb ownership (Makin et al. 2008; Tsakiris 2010; Ehrsson 2012; Blanke et al. 2015). In sum, this work represents the first invasive electrophysiological investigation of the RHI in humans and the findings extend our understanding of the dynamic neural mechanisms that underlie body ownership.

## Materials and Methods

### Participants and Brain Coverage

**Figure 2.**
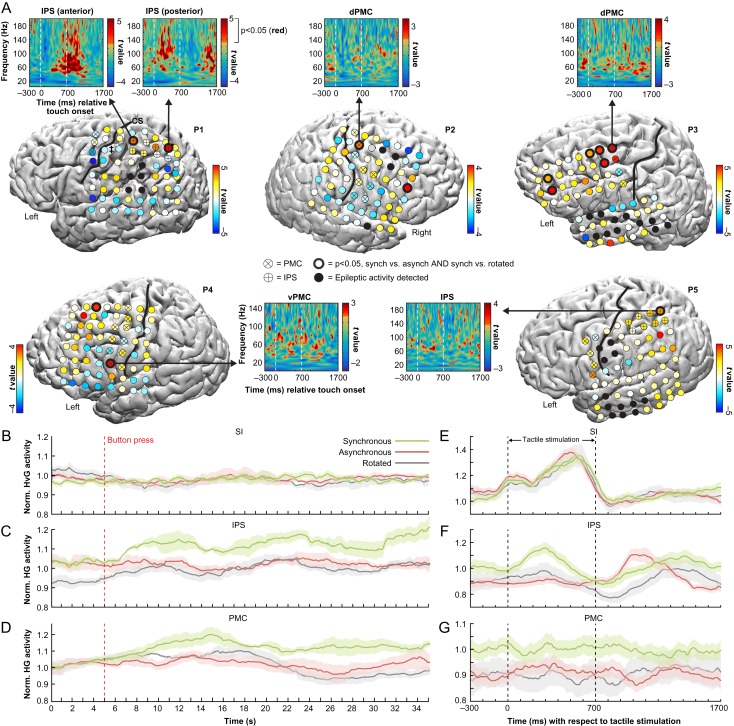
(*A*) ECoG responses during the rubber hand illusion. To identify illusion-specific neuronal population activity, we searched for electrodes that had significantly (*P* < 0.05, FDR-corrected) increased mean high-γ power in the synchronous illusion condition compared with the asynchronous and rotated control conditions. Consistent with our prediction, such electrodes (indicated with bold black circles) were found in all participants and were mainly located in our ROIs in the ventral (v) and dorsal (d) premotor cortex (PMC) and along the intraparietal sulcus (IPS). As seen in the time–frequency *t*-maps showing the *t*-values for the *SynchPOST* versus *AsynchPOST* contrast, this illusion activity was best measured in the high-γ frequency range (70–200 Hz). The electrodes are color coded by their *t* values in the *SynchPOST* versus *AsynchPOST* contrast. (*B*–*D*) High-γ activity over time. The temporal profiles of relative high-γ activity changes for the entire block of stimulation, averaged over trials, are shown for 3 representative electrodes in the SI (see electrode with ^‡^ in P1, panel *A*), IPS (* in P1, panel *A*) and PMC (* in P3, panel *A*) for each of the experimental conditions. As hypothesized, there was a marked high-γ activity increase in the multisensory PMC and IPS—but not in the unisensory SI—in the synchronous condition in association with the button press by which the participants indicated the onset of the illusion. (*E*–*G*) The temporal profiles of relative high-γ activity with respect to the tactile stimulation delivered to the real hand, averaged over all individual touches, for the same representative SI, IPS and PMC electrodes. As expected, in SI, the high-γ activity was directly related to the tactile stimulation and the mean high-γ power across the duration of the epoch did not significantly differ across conditions (*F* = 0.70, *P* = 0.50, one-way ANOVA; panel *E*). Interestingly, the pattern of illusion-related high-γ activity in the IPS (panel *F*) appeared more time-locked to the tactile stimulation than the activity in the PMC (panel *G*). This observation was confirmed in a post hoc analysis across all significant IPS and PMC electrodes (Fig. 3), which suggests distinct functional roles of the PMC and IPS in the limb self-attribution process (see Discussion for details). The shaded areas represent the SEM.

All 5 participants—P1 (33 yo male), P2 (19 yo female), P3 (41 yo male), P4 (13 yo female), and P5 (27 yo male)—were right-handed with a normal IQ based on clinical neuropsychological evaluations. These participants were implanted with 8 × 8 grids of intracranial electrodes that covered the surface of the left temporoparietal (P1), right frontotemporoparietal (P2), left frontotemporal (P3), left frontal (P4), and left frontotemporoparietal lobe (P5) (Fig. 2*A* and S1). The implantation and location of the electrodes were solely determined based on clinical need, and seizure foci were in the left inferior parietal lobe (P1), right medial temporal lobe (P2), left lateral temporal lobe (P3), left anterior, orbital–frontal, and insular lobes (P4), and left medial temporal lobe (P5). Based on previous fMRI findings (Ehrsson et al. 2004), the PMC and IPS bilaterally were defined as regions-of-interest (ROIs) for illusion-related activity. P1 and P5 had electrodes that covered the IPS and all participants had at least 3 electrodes that covered the PMC. In total, 48 of the 288 artifact-free electrodes were defined as belonging to the PMC (32/48) or IPS (16/48) based on their anatomical locations (Fig. 2*A* and S1).

### Experimental Setup

The subjects rested comfortably in their hospital beds, with the head of the bed angled at approximately 45°. A portable screen that was placed on a mobile bedside table was positioned above the subject’s waist. The participants’ hand—the one contralateral to the intracranial electrodes—was positioned behind the screen, hidden from view, while a cosmetic prosthetic hand of the same laterality was placed in front of the screen and was fully visible to the subject (Fig. 1*A*). Thus, a right rubber hand was used for P1, P3, P4, and P5, while a left rubber hand was used for P2. The distance between the index fingers of the real and rubber hands was 15 cm. A piece of white cloth covered the subject’s upper arm to occlude the gap between the shoulder and the prosthetic hand. To induce the RHI (Botvinick and Cohen 1998), a trained experimenter (A.G.) touched the rubber hand and the hidden real hand using 2 identical touch probes that were connected to a biosignal acquisition system and were synchronized with ECoG data acquisition (for details, see ECoG Data Acquisition). The touches were applied to the index, middle, and ring fingers, along the entire length of the fingers, and according to the following regular pattern: index–middle–ring–ring–middle–index–index–middle–ring–ring, and so on. The duration of each touch was 700 ms, and the spacing between the offset of one touch and the onset of the next touch was always 1300 ms (Fig. S2). Consistent with previous studies (Botvinick and Cohen 1998; Ehrsson et al. 2004), we hypothesized that applying spatially and temporally congruent brushstrokes to the rubber and real hands would induce the illusion of owning the rubber hand. To ensure the appropriate timing and duration of the stimuli, the experimenter wore headphones and listened to audio cues that provided the sequence of touches. An analysis of the touch probe data showed that the manually delivered touches were highly accurate in terms of timing (Fig. S3).

### Experimental Conditions and Design

We included 3 experimental conditions: synchronous (illusion) or asynchronous (control) touches on the rubber hand and real hand, or synchronous touches using a rotated (control) rubber hand (Fig. 1*B*). Applying asynchronous touches is an established control condition for disrupting the illusion, which keeps all experimental factors constant except for the temporal congruence of the visual and tactile stimulation (Botvinick and Cohen 1998; Ehrsson et al. 2004). In the asynchronous condition, the onset of the touch on the rubber hand was delayed 1000 ms relative the onset of the touch that was applied to the real hand. Because the duration of each touch was 700 ms and the intertouch interval was 1300 ms, the visual and tactile stimuli did not overlap in this condition. We also included a spatially incongruent rotated condition, which also effectively disrupts the illusion experience (Ehrsson et al. 2004), to control for visual–tactile congruence per se. In this condition, the rubber limb was rotated 180°, with the hand of the rubber limb located in line with the real hand, which was occluded from view behind the screen. Because we aimed to maintain similar visuotactile stimulation with respect to external spatial coordinates, the real hand’s index, middle, and ring fingers were touched in synchrony with the rubber hand’s ring, middle, and index fingers, respectively.

Before commencing the main recording session, we repeated each experimental condition once and quantified the illusion experience using a questionnaire (see Illusion Quantification). The duration of each block was fixed (60 s) and the questionnaire was administered immediately after. Participants were instructed to look at the rubber hand and keep as still as possible during the brushing procedure. The purpose of quantifying the illusion experience prior to the main session was to examine whether participants would experience the illusion in the synchronous condition, which would allow us to instruct them to indicate the illusion onset in the subsequent session. Indeed, all participants experienced the illusion (defined as at least a + 1 rating on statement S1).

In the main recording session, each of the 3 conditions were repeated 4 times in a randomized order. The participants in the synchronous condition were instructed to press a button using the hand that was not stimulated (i.e., the one contralateral to the rubber hand) when they began to experience the illusion, which was defined as the time point when they started to agree with the statement, “It feels as if the rubber hand is my hand.” After the button press, the experimenter continued to brush for 34 s (Fig. 1*D*). In the asynchronous and rotated control conditions, a tone was played after a duration that corresponded to the illusion onset time in the synchronous condition, and the participants were instructed to press the same button in response to hearing the tone.

### Illusion Quantification

The illusion strength was quantified immediately after each experimental condition by asking the subjects to rate 6 statements that were related to their subjective experience on a scale that ranged from −3 (“I disagree completely”) to +3 (“I agree completely”) (Longo et al. 2008; Guterstam and Ehrsson 2012; Guterstam et al. 2011, 2013, 2016). The statements were adopted from the original RHI questionnaire (Botvinick and Cohen 1998) and featured 3 illusion and 3 control statements (Table 1). The illusion statements (S1–S3) were designed to reflect the key elements of the illusion experience, while the purpose of the control statements (S4–S6) was to account for suggestibility and task compliance. In our statistical analysis, we used the average rating of the illusion and control statements as inputs in our model. Because the rating averages were normally distributed for all of the experimental conditions (using the Kolmogorov–Smirnov test) and parametric analysis of ordinary averages of Likert scale data is justifiable by the Central Limit Theorem, we analyzed the questionnaire data with a 2 × 3 ANOVA with the factors statement type (illusion, control) and condition (synchronous, asynchronous, rotated). The questionnaire results are shown in Figure 2*C*.

**Table 1:** Questionnaire statements.

S1	It felt as if the rubber hand were my hand.
S2	It seemed as if I were feeling the touch of the paintbrush in the location where I saw the rubber hand touched.
S3	It seemed as though the touch I felt was caused by the paintbrush touching the rubber hand.
S4	It felt as if my (real) hand were drifting towards the left (towards the rubber hand).
S5	It felt as if I had 2 right hands/arms.
S6	It seemed as if the touch I was feeling came from somewhere between my own hand and the rubber hand.

### Cortical Reconstructions and Electrode Overlay

We generated the cortical reconstructions and electrode overlays based on previously published procedures (Wander et al. 2013). In short, postoperative, clinically indicated, computed tomography scans (1 mm resolution) were coregistered with the preoperative structural T1-weighted MRI scans (3-D MPRAGE sequence, voxel size = 1 mm^3^, field-of-view = 256 mm × 256 mm, 170 slices, repetition time = 1900 ms, echo time = 3 ms, flip angle = 8°) using SPM8 (The FIL Methods group, London, UK). Reconstructions of the cortical surface were generated with FreeSurfer (Martinos Center for Biomedical Imaging, Boston, MA, USA) and custom MATLAB (MathWorks, Natick, MA, USA) code. Projections of the electrode grids relative to the surface cortical structures were created as described by Hermes et al. (2010) (see Fig. 2 for the results).

### ECoG Data Acquisition

All 5 participants were implanted with an Ad-Tech (Racine, WI) 64-contact subdural electrode array with 4-mm contacts, 2.4-mm-diameter exposed recording surfaces, and 10-mm contact spacing in an 8 × 8 rectangular array. Implantations were performed at Harborview Medical Center (Seattle, WA, USA) for P1, P2, P3, and P5 and Seattle Children’s Hospital (Seattle, WA, USA) for P4. Recordings were performed at the patients’ bedsides without interrupting the clinical recording. Cortical potentials were referenced against a scalp electrode.

The electrophysiological recordings were performed using the Tucker-Davis Technologies (TDT, Alachua, FL) biosignal acquisition system, which consists of the following components: an RZ5D BioAmp Processor, a PZ5 NeuroDigitizer, and a LZ48 Battery Pack. The recording circuits were programmed with the TDT Real-Time Processor Visual Design Studio (RPvdsEx). The circuits were loaded to the processor, and signals were acquired at run-time with the TDT OpenEx application. Neurophysiologic signals were acquired and stored with a sampling rate of 1220 Hz without any preprocessing. Programmable run-time parameters and the brushstroke data from the custom-built touch probe (Karolinska Institutet, Stockholm, Sweden), which registered the onset and offset for each touch, were synchronously stored at 1220 Hz (for details on the touch probe hardware and validation testing, please see (Collins et al. 2017)). The ECoG data for P4 were recorded using the g.USBamps (G.TEC Medical Engineering GMBH, Austria) biosignal acquisition system sampled at 1200 Hz and were digitized and processed using the BCI2000 software (Schalk et al. 2004). Because a severe nonphysiological artifact was detected at approximately 180–220 Hz, only frequencies ≤150 Hz were analyzed for this subject.

### ECoG Data Preprocessing

Data preprocessing was performed in MATLAB and included manually removing nonphysiological artifacts, epileptic activity and noise. To reject common-mode noise, all ECoG channels were re-referenced to a common average reference. The notch filter from the MATLAB Signal Processing Toolbox was used to remove 60 Hz noise and its second and third harmonics. To further reduce noise, signals were high-pass filtered at 3 Hz and low-pass filtered at 500 Hz using fourth order Butterworth filters.

For the main analyses (Figs 2*A*,*E*–*G* and 4*C*,*D*), the signals were segmented into 2000-ms-long epochs that were aligned with the onset of the touches that were delivered to the real hand, as measured by the touch probe. Specifically, the epochs began 300 ms before the onset of the touch and ended 1700 ms after the touch onset (i.e., 1000 ms after touch offset). Because the participants indicated the illusion onset with a button press—or, in the control conditions, pressing a button in response to a tone after the corresponding duration of time—each epoch was labeled as belonging to one of the following 6 conditions: *SynchPRE*, *SynchPOST, AsynchPRE*, *AsynchPOST, RotatedPRE*, and *RotatedPOST* (“pre” and “post” indicated before and after the button press). The epoch that coincided with the button press was disregarded to prevent motor related activity affecting the subsequent data analysis. The justification for dividing the signals into 2000-ms-epochs was supported by 2 main arguments: 1) it allowed us to examine the temporal relationship between illusion-related activity and tactile stimulation, which is an outstanding question in the fMRI literature on the RHI; and 2) it increased the statistical power for our main analyses, which constituted a potential severe issue because we were only able to repeat each condition 4 times due to time limitations related to patient fatigue and a typical RHI onset is relatively long (10–20 s) (Ehrsson et al. 2004; Ehrsson 2012).

For the connectivity analysis (Fig. 5), the signals were segmented into epochs that represented all of the touches before the button press (pre epochs) and all of the touches after the button press (post epochs). The beginning and end of the pre epochs were defined by the onset of the first touch of the repetition and the offset of the last touch before the participant pressed the button, while the start and end of the post epochs were defined by the onset of the first touch after the button press and the offset of the last touch of the repetition. In cases where the button press occurred during a touch event, that touch was disregarded to prevent motor activity from affecting the connectivity analysis. The approach of using longer epochs for the connectivity analysis was supported by the nature of the cross-correlation analysis, see the ECoG Connectivity Analysis.

### ECoG Regional Analysis

For the main analysis (Fig. 2*A*), we used both the Hilbert transform and a wavelet approach to construct time–frequency dynamic spectra. For the Hilbert transform approach, signals were band-pass filtered for the frequency band of interest (high-gamma, 70–200 Hz for all participants except P4, in which high-γ was defined as 70–150 Hz due to artefacts in the 180–220 Hz range), as well as the α (8–12 Hz), β (12–24 Hz), and γ bands (30–60 Hz). An estimate of the average band power for each epoch was calculated using the square of the magnitude of the Hilbert transform. To identify illusion-related activity, in a within-subject approach, we compared the mean high-γ power for the *SynchPOST* compared with the *AsynchPOST* epochs for all electrodes using unpaired 2-tailed *t*-tests. To control for multiple comparisons within our ROIs, we used the Benjamini–Hochberg step-up procedure to control the false discovery rate (FDR) across the total number of ROI electrodes within each subject. Outside of the ROIs, we applied FDR-correction across all artifact-free grid electrodes. To exclude the possibility that activity observed in the *SynchPOST* versus *AsynchPOST* contrast were not related to the illusion experience, but rather to the visuotactile synchrony per se, we contrasted the *SynchPOST* versus *RotatedPOST* epochs using the same statistical approach (Gentile et al. 2013; Guterstam et al. 2013). The results are presented for all electrodes within each participant (Fig. 2*A*): the color codes for the electrodes represent the *t* values for the *SynchPOST* versus *AsynchPOST* contrast and a bold circle indicates the electrode(s) in which both contrasts (*SynchPOST* vs. *AsynchPOST* and *SynchPOST* vs. *RotatedPOST*) were statistically significant. Thus, the bold circled electrodes represent areas in which there was illusion specific high-γ activity. The alpha level was always set to 0.05.

To complement the Hilbert transform analysis, the wavelet approach used a Morlet wavelet (Goupillaud et al. 1984) to convolve with the voltage time-series of each epoch to generate a time–frequency estimate for every frequency bin between 3 and 200 Hz, which allowed for an investigation of the changes in the power spectrum density across all frequencies in relation to the timing of the tactile stimulation. For the electrodes in which we observed illusion specific activity using the above Hilbert transform approach, we calculated significant changes in power across time bins and frequencies by comparing (unpaired 2-tailed *t*-tests) the power density maps (with a pixel-per-pixel approach) that belonged to the *SynchPOST* versus *AsynchPOST* epochs (Miller et al. 2007). This comparison generated a time–frequency map of *t* values (Fig. 2, see cut-outs), in which pixels that had significant power increases (*P* < 0.05, uncorrected) are colored red, for display purposes only. Two lines are overlaid on the *t*-map to indicate the onset and offset of the tactile stimulation to the real hand.

### High-γ Activity in IPS and PMC in Relation to Tactile Stimulation

To examine illusion-related high-γ activity in the IPS and PMC in relation to the applied tactile stimulation, we segmented the 2000-ms epochs of data (Fig. 1*E*) into 2 periods: one 700-ms “during touch” period that corresponded to the tactile stimulation of the real hand as measured by the touch sensor, and one 1300-ms “between touches” period that corresponded to the time between the offset of one touch and the onset of the next touch. Then, we estimated the mean high-γ power for the “during touch” and “between touches” periods across all epochs in the *SynchPOST* and *AsynchPOST* conditions for all IPS and PMC electrodes that showed significant illusion-related high-γ activity in our main analysis (Fig. 2A). The high-γ power was normalized relative to the mean high-γ power across all epochs and conditions within each participant to accommodate for baseline differences in the level of high-γ signal between individuals. First, we investigated the effect of visuotactile synchrony for the “during touch” and “between touches” periods separately in the PMC and IPS. Because the data sets had unequal variances and did not meet the assumptions for an ANOVA, we used a permutation testing approach in which the labels for the “*SynchPOST*” and “*AsynchPOST*” epochs were permuted. Second, we examined whether the illusion-related high-γ activity was differently modulated by the delivered tactile stimulation in the IPS compared with the PMC. To identify high-γ activity that is specific to the cortical area (IPS/PMC), the period (during touch/between touches) as well as the illusion experience, the critical analysis is the following 3-way interaction: area (IPS, PMC) × period (during touch, between touches) × synchrony (*SynchPOST*, *AsynchPOST*). In this analysis, we used a permutation testing approach in which the labels of the “during touch” and “between touches” epochs were permuted (10 000 iterations) to calculate the *P* value for the interaction term ([IPS > PMC] vs. [during > between touches] vs. [SynchPOST > AsynchPOST]). In addition, because peripheral tactile stimulation takes approximately 200 ms to activate multisensory neurons in the IPS (Duhamel et al. 1998), we estimated the 3-way interaction for a range of different forward-shifts of in the 700-ms-long data window that corresponded to the “during touch” period. Thus, the data window of ECoG data that was defined as “during touch” was continuously shifted relative to the touch onset that was measured by the peripheral touch sensor, and for every shift of the “during touch” data window, the “between touches” data window correspondingly shifted, so that the data window durations were always 700 and 1300 ms, respectively.

### ECoG Connectivity Analysis

To examine the dynamic interplay between SI and IPS in relation to the illusion, we examined connectivity changes to SI in participant P1, who was the only participant with appropriate parietal electrode coverage who had participated in a sensory stimulation screening to identify SI proper. The sensory stimulation screening was conducted for clinical purposes and identified electrode #47 as the SI representation for the right middle finger. Thus, this electrode was used as seed. Then, we calculated the cross-correlation coefficient (Pearson’s *r*) in the *SynchPOST* condition between the time series of activity in the SI electrode and the activity in all of the other electrodes, which were shifted in time from 0 ms to +250 ms using 1-sample (1/1220 = 0.82 ms) steps. Notably, here we used the time series of activity for the entire period after the illusion onset (i.e., we did not segment the data into 2000-ms epochs). Then, we calculated the *Z* value for each *r* coefficient using the Fisher *r*-to-Z-transformation. To control for spatial autocorrelations and identify interelectrode correlations that were specific to the illusion, we computed the correlation difference between the *SynchPOST* and *SynchPRE* conditions for each electrode and time lag. Because the period before and after the illusion onset are matched in all aspects, including visuotactile synchrony per se, any significant correlation increase in this analysis must be related to the illusion experience. We calculated the *Z* value for the difference in correlations between *SynchPOST* and *SynchPRE* for each channel and time lag (Fig. 5*A*) and normalized this value for the largest correlation difference. We assessed statistical significance by calculating the critical *Z* for achieving *P* = 0.05 (normalized *Z* = 0.24), corrected for multiple comparisons using the Bonferroni correction across all 64 channels and 305 time lags. It should be noted that we used the more conservative Bonferroni correction instead of the FDR correction because of the exploratory nature of this analysis. Furthermore, to control for non-specific time effects, we repeated the above analysis with the *asynchronous* control condition, that is, estimating the correlation difference between *AsynchPOST* compared with *AsynchPRE* (Fig. 5*B*).

To examine the nature of the *SynchPOST* versus *SynchPRE* correlation difference between the seed electrode in SI and the posterior IPS (electrodes #47 and #50; Fig. 5*A*), we plotted the SI–IPS correlation coefficient over time (i.e., for time lags 0–250 ms of the IPS signal) separately for the *SynchPRE* and *SynchPOST* conditions (Fig. 5*C*). To control for unspecific time effects, we repeated the analysis for the *asynchronous* condition (Fig. 5*D*). Because the time lag of 200 ms had the greatest correlational difference, we ran a Bode plot analysis for this time lag in the *SynchPRE* and *SynchPOST* conditions (Fig. S4). This analysis informs the magnitude and phase shift of the frequency responses of the SI signal (input) relative the IPS signal (output). Examining the Bode function differences between *SynchPRE* and *SynchPOST* reveal information about the band spectra that drive the correlation difference.

### Functional MRI Experiment

The experimental conditions and design of the fMRI experiment were identical to the ECoG experiment. The acquisition, preprocessing and statistical analysis of the fMRI data followed standard procedures and were in accordance with previous published fMRI studies on the RHI (Ehrsson et al. 2004; Guterstam et al. 2013). Please see Supplementary Materials and Methods for detailed information about the fMRI methods.

## Results

### Behavioral Results

Before examining the ECoG and fMRI results related to the RHI, we tested whether the experimental setup successfully manipulated the sense of limb ownership at the behavioral level in our participants. To this end, we analyzed the data from a questionnaire experiment conducted immediately before the ECoG recording session, in which participants rated 6 statements (Table 1) that were related to their subjective illusion experience on a scale that ranged from −3 (completely disagree) to +3 (completely agree). All 5 participants positively rated the key ownership statement S1 (“It felt as if the rubber hand were my hand.”) in the synchronous condition (median = 2; range: 1–3), which was consistently lower than in the asynchronous (all −3) and rotated conditions (median = −3; range: −3 to 2). These results demonstrated that the illusion was successfully elicited in the synchronous condition in all individuals (Fig. 1C). At the group level, we compared the mean ratings of the illusion (S1–S3) and control statements (S4–S6) using a 2 × 3 ANOVA with factors statement type (illusion, control) and condition (synchronous, asynchronous, rotated). The results showed that participants rated the illusion statements significantly higher than the control statements (main effect of statement type: *F*_1,4_ = 110.2, *P* < 0.001) in the synchronous, but not in the asynchronous and rotated conditions (main effect of condition: *F*_2,8_ = 17.5, *P* = 0.001; interaction statement type × condition: *F*_2,8_ = 14.1, *P* = 0.002), which confirmed that the RHI depends on temporally and spatially congruent visuotactile stimulation.

### High-γ Activity in IPS and PMC Reflects the RHI

The main analysis focused on the high-γ response in the period after the illusion onset, which participants indicated with a button press, in the synchronous condition compared with the corresponding periods in the asynchronous and rotated control conditions. To increase the statistical power and investigate the precise temporal relationship between the illusion-related activity and the applied tactile stimulation, the ECoG data were segmented into 2000-ms epochs that were aligned with the onset of the touch that was delivered to the real hand and was measured by the touch probe (Fig. 1*D*,*E*). The epochs were labeled as belonging to 1 of 6 experimental conditions: *SynchPRE*, *SynchPOST, AsynchPRE*, *AsynchPOST, RotatedPRE*, and *RotatedPOST*, where “pre” and “post” indicates before and after the button press. To identify illusion specific high-γ activity, we evaluated the average high-γ power for each epoch and searched for electrodes that displayed significant increases in power in the *SynchPOST* condition compared with the *AsynchPOST* and *RotatedPOST* control conditions using 2 separate *t*-tests. We employed the FDR correction to control for multiple comparisons within each participant, using the PMC and IPS electrodes as search space within our anatomically predefined ROIs, and all electrodes as search space for regions outside the ROIs. The results showed that 13 of the 288 electrodes showed significant illusion-specific high-γ responses (Table S1), of which 6 were located in our ROIs in the IPS (P1 and P5) and PMC (P2–P4) (Fig. 2*A*). Five of the significant ROI electrodes also survived the correction for multiple comparisons across all electrodes, which is more than is expected by chance (*P* = 0.033, permutation test with 10 000 iterations) and thus consistent with our prediction that the PMC and IPS support body ownership. In addition to the high-γ band, we evaluated activity in the α- (8–12 Hz), β- (12–24 Hz), and γ-bands (30–60 Hz) in our ROIs. Apart from one electrode in the PMC (#8 in P3, see Fig. 2*A* and S1) that had illusion-specific high-γ activity as well as significant γ-band activity (*P* < 0.05, FDR-corrected), none of the PMC and IPS electrodes had power increases in the α-, β-, or γ-bands that even demonstrated a trend towards statistical significance (all *P* > 0.10, FDR-corrected). These findings are supported by the visual inspection of the time–frequency plots that show the *t* value for the *SynchPOST* versus *AsynchPOST* contrast for each frequency and time bin (Fig. 2*A*, see cut-outs), which indicate that the illusion-specific responses are primarily concentrated in the higher frequency bands (>60 Hz). Finally, in a supplementary analysis we compared the high-γ activity recorded after the onset of the illusion with that recorded before it commenced. Four out of 5 participants showed increased high-γ activity in the post compared with preillusion onset period that was specific to the synchronous condition and spatially overlapped with the results of the main analysis described above (Fig. 2*A*), which further strengthen the conclusion that high-γ activity in the PMC and IPS support limb ownership (for detailed results and discussion, see Table S3).

### High-γ Activity in IPS and PMC in Relation to Tactile Stimulation

**Figure 3.**
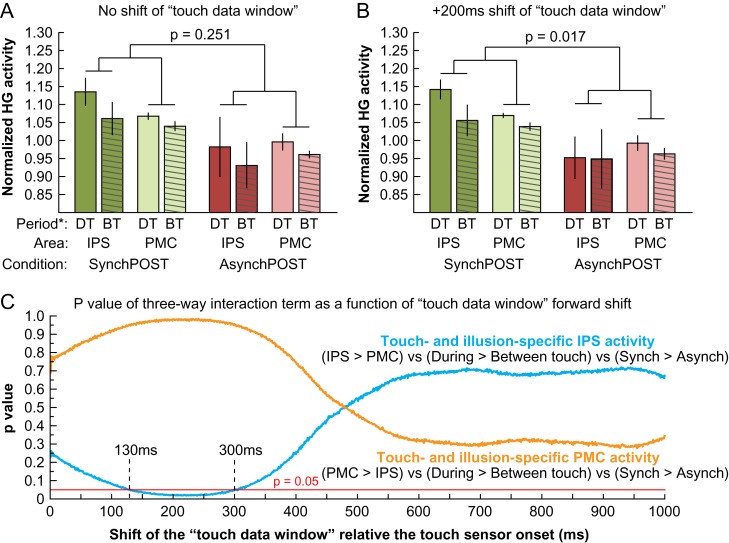
Neural activity in IPS and PMC in relation to tactile stimulation during the RHI. To examine the temporal profiles of the illusion-related high-γ activity observed in the IPS and PMC (Fig. 2) in relation to the applied tactile stimulation, we segmented the ECoG data into “during touch” (DT; 700-ms data window) and “between touches” periods (BT; 1300-ms data window) and compared the mean high-γ power across the 2 period types (DT, BT), cortical areas (IPS, PMC), and visuotactile synchrony (*SynchPOST*, *AsynchPOST*). To examine the hypothesis that illusion-related activity in the IPS is more time-locked to the tactile stimulation than the PMC, the analysis-of-interest was the 3-way interaction between area × period type × synchrony. Because it typically takes peripheral tactile stimulation about 200 ms to activate neurons in multisensory areas (Duhamel et al. 1998), we shifted DT and BT data windows in the ECoG signal 200 ms forward in time relative the peripheral touch sensor. The results showed that a 200 ms shift of the “touch data window” yielded a significant 3-way interaction (*B*), which was not the case without a shift (*A*). Analyzing the 3-way interaction term over continuous shifts in the “touch data window” revealed that the illusion-related IPS activity was significantly more modulated by the tactile stimulus than PMC for shifts between 130 and 300 ms (*C*), which is in accordance with temporal response properties of IPS neurons to tactile stimulation (Duhamel et al. 1998). We speculate that this pattern of results may reflect different functional roles of these 2 key areas in the limb self-attribution process, in which the IPS is more involved in the self-attribution of sensory signals that originate from the “owned” rubber hand, while the PMC—as the hierarchically highest level of multisensory body representation that is targeted by the RHI—is primarily involved in generating the continuous feeling of ownership. *DT = During touch; BT = Between touches.

To examine the high-γ activity in the IPS and PMC with respect to the applied tactile stimulation, we segmented the 2000-ms epochs of data (Fig. 1*E*) into one 700-ms “during touch” period corresponding to the tactile stimulation of the real hand as measured by the touch sensor and one 1300-ms “between touches” period that corresponded to the time in-between 2 sequential touches. The mean high-γ power for the “during touch” and “between touches” periods were then extracted for the *SynchPOST* and *AsynchPOST* conditions for all IPS and PMC electrodes that displayed significant high-γ activity in our main analysis (Fig. 2*A*). Using a permutation testing approach (see Materials and Methods for details), we first analyzed the effect of visuotactile synchrony. The results showed that there was a significant effect of visuotactile synchrony for both the “during touch” and “between touches” periods in the IPS as well as the PMC (all *P* < 0.001, permutation testing with 10 000 iterations; Fig. 3*A*,*B*), which suggests that the RHI is associated with a continuously elevated level of neural activity in these 2 areas both during and in-between individual touches. Second, we examined whether the illusion-related high-γ activity was differently modulated by the delivered tactile stimulation in the IPS compared with the PMC. To this end, we estimated the key 3-way interaction between cortical area (IPS, PMC) × period (during touch, between touches) × synchrony (*SynchPOST*, *AsynchPOST*). Furthermore, because it takes approximately 200 ms for peripheral tactile stimulation to activate multisensory IPS neurons (Duhamel et al. 1998), we estimated the 3-way interaction term for a range of different forward-shifts in time using a sliding window approach, in which the windows of ECoG data labeled “during touch” and “between touches” were shifted relative to the touch onset as measured by the touch sensor. The results showed that illusion-specific touch-associated high-γ activity did not significantly differ between IPS and PMC when the touch data window remained unshifted relative to the onset of the touch sensor (*P* = 0.251, Fig. 3*A*). However, when it was shifted 200 ms forward in time (Duhamel et al. 1998), the illusion-specific high-γ activity in the IPS compared with the PMC was significantly modulated by tactile stimulation (*P* = 0.017), which was driven by an increased during-versus-between-touches difference in the *SynchPOST* and a corresponding decrease in the *AsynchPOST* condition (Fig. 3*B*). Plotting the p value for the 3-way interaction term as a function of the touch data window time shift (0–1000 ms) showed that the illusion-specific high-γ activity in the IPS was significantly (*P* < 0.05) modulated by tactile stimulation for shifts between 130 and 300 ms (Fig. 3*C*; blue curve), which is compatible with the known temporal response properties of IPS neurons to tactile stimulation (Duhamel et al. 1998). In contrast, the illusion-specific high-γ activity in the PMC was not significantly modulated by tactile stimulation for any time shift of the touch data window (Fig. 3*C*; orange curve). Together, these findings suggest that ownership-related increases in activity in neuronal populations in both the IPS and PMC are sustained during and in-between individual touches, but that the activity in IPS is significantly more strongly coupled with the processing of tactile stimulation.

### Overlapping fMRI and ECoG Activity in the PMC and IPS

**Figure 4.**
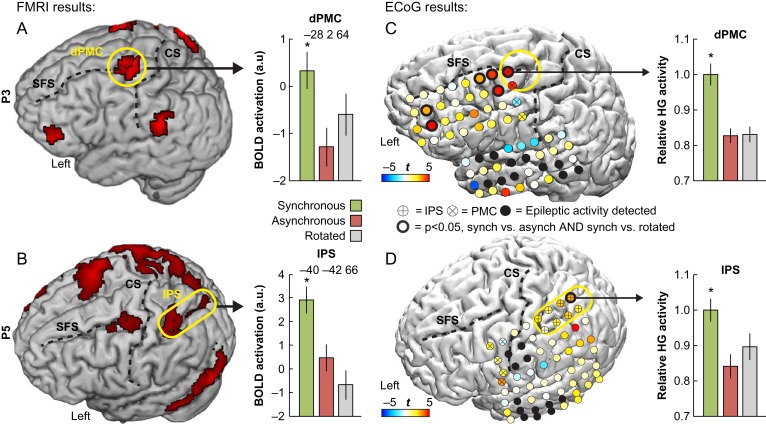
Overlap between ECoG and BOLD responses. Two participants (P3 and P5) underwent fMRI prior to the ECoG grid implantation, which allowed for a direct comparison of BOLD and high-γ responses related to the RHI. Consistent with our hypothesis, there were overlaps of significant activity in the left dPMC (P3: panels *A* and *C*) and in the left IPS (P5: panels *B* and *D*), which suggests that illusion-related hemodynamic BOLD responses in these regions reflect high-γ activity in neuronal populations. The high-γ activity is normalized relative to the largest response. The yellow circles show regions of overlap between ECoG and BOLD activity within our ROIs. For visualization purposes, the BOLD activation maps are thresholded at *P* < 0.05 (uncorrected). CS = central sulcus; SFS = superior frontal sulcus; HG = high-γ. *P < 0.05, corrected.

Prior to electrode grid implantation, 2 participants (P3 and P5) completed a blocked-design fMRI experiment that featured experimental conditions that were identical to those in the subsequent ECoG experiment, and allowed for the descriptive comparison of activation maps across imaging modalities. To identify illusion specific BOLD responses, we contrasted synchronous versus asynchronous (*P* < 0.01, uncorrected) and used the synchronous versus rotated contrast as an inclusive mask (thresholded at *P* < 0.05, uncorrected), which is consistent with the ECoG analysis approach and previously published fMRI studies on the RHI (Gentile et al. 2013; Guterstam et al. 2013). In line with our hypotheses, the results showed that the illusion experience was associated with increased BOLD activity in the bilateral PMC (P3 and P5) and along the right (P3) and bilateral IPS (P5) (Table S1). In P3, who had left frontotemporal electrode coverage, we observed significant BOLD activity in the left dPMC (*P* = 0.045, corrected; Fig. 4*A*) that overlapped with illusion-specific high-γ activity (*P* < 0.05, corrected; Fig. 4*C*). In P5, whose electrode grid covered the left temporoparietal lobes, we found overlapping BOLD (*P* = 0.037, corrected; Fig. 4*B*) and high-γ activity (*P* < 0.05, corrected; Fig. 4*D*) in the left IPS. These findings suggest that ownership-related BOLD responses in premotor–intraparietal areas (Ehrsson et al. 2004) reflect a local increase in the average neuronal population firing (Ray and Maunsell 2011).

We also observed illusion-related BOLD activations in the supramarginal gyrus (P3 and P5), lateral cerebellum (P3), and lateral occipital cortex (P5); regions that are outside the premotor–intraparietal cortices and have been associated with the RHI in fMRI studies (Table S1), which were not covered by the electrode grids (Ehrsson et al. 2004; Guterstam et al. 2013; Limanowski and Blankenburg 2016). Interestingly, P5 had much greater illusion-related BOLD activity in the PMC and IPS in the right hemisphere (Table S1) that was contralateral to the ECoG grid, which might explain why only one electrode had significant high-γ activity in this participant (Fig. 4*D*).

### Ownership-Specific Connectivity From the SI to the IPS

**Figure 5.**
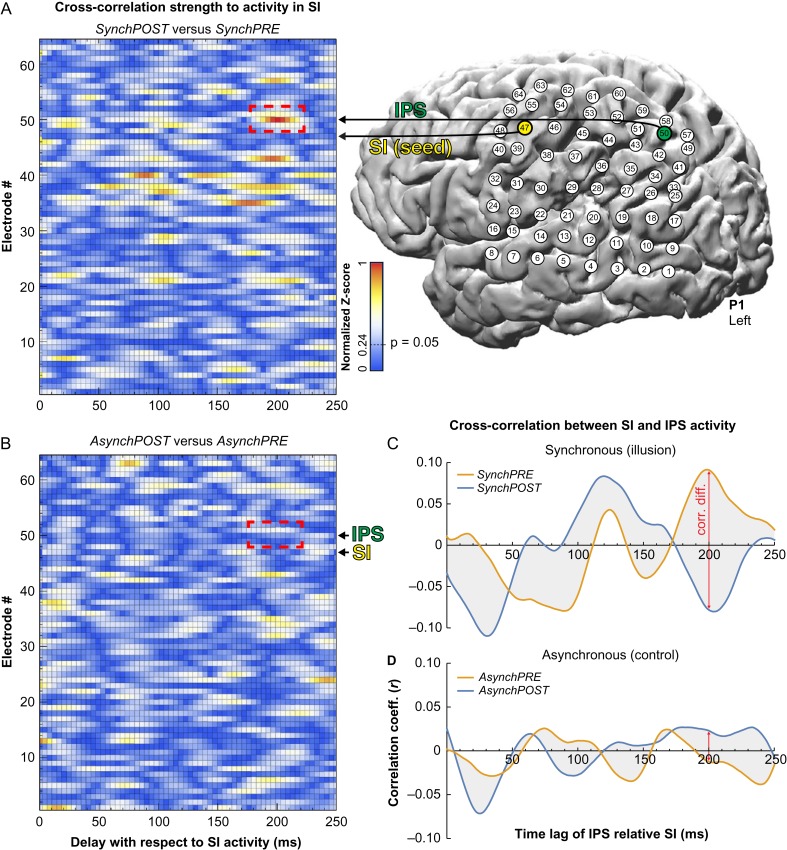
Connectivity from hand-SI to IPS during the RHI. (*A*) We examined illusion-related changes in the flow of information from the representation of the hand in SI, which was identified by cortical stimulation, to the parts of the parietal and temporal lobes that were covered with electrodes in P1. For each electrode across the grid, the times series of activity before and after the illusion onset was extracted and shifted up to 250 ms relative to the SI activity. To identify illusion-specific connectivity changes, we searched for electrodes in which the cross-correlation strength with SI activity was significantly stronger after (*SynchPOST*) compared with before the illusion onset (*SynchPRE*). We found that the largest correlation difference occurred in electrode #50 (indicated by a dashed red box) and adjacent electrodes (#43 and #57) in the posterior IPS, when the IPS signal was delayed 200 ms relative to the SI signal (all *P* < 0.05, corrected). Thus, SI activity significantly predicted IPS activity 200 ms later during the illusion. (*B*) In the asynchronous control condition, there was no significant correlation difference for the corresponding electrode and time lag (*P* > 0.05, corrected; see dashed red box), which excludes potential confounds that are related to the general effects of time. (*C*, *D*) Analyzing the SI–IPS correlation coefficient as a function of the time lag of the IPS signal showed that the correlation difference at 200 ms is driven by a strong positive correlation in the *SynchPRE* and an equally strong negative correlation in the *SynchPOST* condition (*C*). This difference was not observed in the *asynchronous* control condition (*D*). Taken together, these findings show that SI influences IPS activity before and after the illusion onset, but that the nature of the influence dramatically changes after the illusion onset, which reveals a neural mechanism for how low-level tactile signals are integrated into higher-order body representations in the IPS to shape the feeling of limb ownership.

In the process of attributing ownership to the rubber hand, the brain must combine tactile information from the real hand—processed by the hand section of the primary somatosensory cortex (hand-SI)—with visual information from the rubber hand being touched, which is processed by the primary visual areas. We hypothesized that the IPS plays a key role in this visuotactile integration process because it has strong anatomical connections to both SI and early visual areas (Andersen et al. 1990; Gallese et al. 1994), features neurons that integrate visual and tactile signals within peripersonal space (Graziano 2000) and shows increased BOLD activity during the RHI (Ehrsson et al. 2004). However, due to the limited temporal resolution of fMRI, little is known about this neuronal interplay between hand-SI and IPS that underlies the emergence of the illusion. Here, we made use of cortical stimulation to localize hand-SI with high precision and exploited the high temporal resolution of ECoG to examine the flow of information from the hand-SI to the temporal and parietal lobes in one participant with appropriate electrode coverage (P1). Specifically, we extracted the time series of cortical surface potentials for the entire periods before and after the illusion onset (i.e., the data were not segmented into 2000-ms-epochs for this analysis; see Methods section for details) for each electrode across the grid in the synchronous condition, and shifted the activity time series up to 250 ms using 1-sample (1/1220 = 0.82 ms) steps relative to the SI signal. To identify illusion-specific connectivity changes from hand-SI, we searched for electrodes in which the cross-correlation strength to the SI signal was significantly different after compared with before the illusion onset (*SynchPOST* vs. *SynchPRE*) (Fig. 5*A*). The results showed that the largest difference in cross-correlation occurred in the IPS electrode #50 (normalized *Z* = 1.0, *P* < 0.05, corrected) and its surrounding electrodes in the IPS (#43: normalized *Z* = 0.92, *P* < 0.05, corrected; and #57: normalized *Z* = 0.80, *P* < 0.05, corrected), when their activity time series were shifted approximately 200 ms relative the hand-SI activity (Fig. 5*A*). To exclude potential general effects of time, we repeated the analysis in the asynchronous control condition (i.e., comparing *AsynchPOST* vs. *AsynchPRE*), but found no significant correlation difference from SI to the IPS (normalized *Z* = 0.21, *P* > 0.05; Fig. 5*B*, see the red dashed box). These findings suggest that activity in hand-SI significantly predicts IPS activity 200 ms later during the illusion.

To explore the driving factors behind the observed correlation difference between *SynchPOST* and *SynchPRE* for the peak IPS electrode (#50; Fig. 5*A*), we examined the correlation between the SI and IPS signals over time for the *SynchPRE* and *SynchPOST* conditions individually. As shown in Figure 5*C*, the correlation difference revealed at around 200 ms was driven by a strong positive correlation in *SynchPRE* and an equally strong negative correlation between the SI and IPS signals in *SynchPOST*, which were not observed in the asynchronous control condition (Fig. 5*D*). A strikingly similar pattern of results was observed in the surrounding posterior parietal electrodes that also showed large *SynchPOST* versus *SynchPRE* cross-correlation differences at around 200 ms (specifically #57, #43, and #35; see Figs 5*A* and S5), suggesting that the change of sign of the cross-correlation in the peak IPS electrode (#50) reflect an aspect of the underlying neural signal processing in the posterior parietal cortex rather than being a statistical anomaly. An analysis of the magnitude and phase shift of the frequency responses for *SynchPRE* and *SynchPOST* at the 200 ms time lag (in electrode #50) showed that this difference was primarily driven by 1 lower frequency (<40 Hz) and 2 higher frequency (90–110 and 130–150 Hz) signal components of opposite phases, as illustrated by a Bode plot analysis (Fig. S4). These findings suggest that SI influences IPS activity with a 200 ms delay across α-, β-, and high-γ frequency ranges during the entire block of visuotactile stimulation; however, the nature of the influence changes at the onset of the illusion.

## Discussion

We used the RHI in combination with ECoG and fMRI to investigate the neural mechanisms that underlie the feeling of limb ownership. Our results revealed 3 main novel findings. First, we found that high-γ activity, which is a reliable proxy of the local average neuronal firing rate (Ray et al. 2008; Ray and Maunsell 2011; Miller et al. 2014; Suffczynski et al. 2014), in the premotor and intraparietal cortices reflects the sense of ownership of the artificial limb. Second, the same set of areas also showed increased fMRI-BOLD responses during the illusion, directly linking ownership-related BOLD responses and local neuronal population firing. Finally, we found that the temporal profile of high-γ activity in the IPS was more related to the applied tactile stimulation than the PMC, which showed a more sustained high-γ response during the illusion, suggesting different functional roles of the premotor and intraparietal cortices in generating and sustaining the RHI. Taken together, these results suggest that ownership of a seen limb is reflected in neuronal population firing in premotor–intraparietal areas and shed light on the dynamic neural interplay between primary sensory and multisensory areas in the process of attributing ownership to one’s limbs.

The experience of the RHI was consistently coupled with high-γ activity in multisensory areas in the premotor cortex and along the IPS (Fig. 2*A*). These results are highly compatible with previous studies showing that the intracranial high-γ signal represents the best metric for localized cortical activation (Crone et al. 1998; Lachaux et al. 2005; Dastjerdi et al. 2013; Johnson and Knight 2015) and correlates with the BOLD signal (Logothetis et al. 2001; Conner et al. 2011; Esposito et al. 2013; Hermes et al. 2017), whose peaks of activation during the RHI have been consistently localized to the premotor and intraparietal cortices in fMRI studies (Ehrsson et al. 2004; Brozzoli et al. 2012; Guterstam et al. 2013; Limanowski and Blankenburg 2016). These areas are considered prime candidates for mediating the feeling of body ownership (Makin et al. 2008; Tsakiris 2010; Ehrsson 2012; Blanke et al. 2015; Kilteni et al. 2015), based on the fMRI evidence from the RHI (Ehrsson et al. 2004; Brozzoli et al. 2012; Guterstam et al. 2013) as well as invasive animal studies showing that premotor–intraparietal neurons integrate visual, tactile and proprioceptive information and are involved in constructing multisensory representations of the body and its surrounding peripersonal space (Graziano et al. 1997; Graziano 2000; Graziano and Botvinick 2002). Intriguingly, one study exposed a macaque to the RHI while recording from single neurons in the anterior bank of the IPS of parietal area 5 (Graziano 2017) and showed that 4 out of 5 tested neurons responded as if the (supposedly) owned artificial arm were the monkey’s own arm (Graziano 2000). However, invasive neurophysiological studies of the RHI have been lacking in humans, who have the advantage of being able to report their subjective experiences. Thus, the present results constitute the first invasive evidence in humans that neuronal populations in premotor and intraparietal cortices respond selectively to the feeling of ownership of a seen hand. In conjunction with the observed overlap between ownership-related high-γ activity and BOLD responses, this study bridges previous invasive animal and noninvasive human neuroimaging findings.

The presence of consistent illusion-specific activity in the high-γ band, and the absence of such activity in the α-, β-, or γ-bands in our data highlight the importance of employing intracranial ECoG in addition to scalp EEG. While ECoG is ideally suited for capturing high-γ band oscillations (Ritaccio et al. 2014), scalp EEG suffers substantially lower signal-to-noise ratio in this band spectra due to artifact contamination from muscle activity in higher frequencies with lower amplitudes (Goncharova et al. 2003; Witham and Baker 2007). These factors might explain the inconsistent results among previous scalp EEG studies on the RHI, which focused on either somatosensory event-related potentials (Peled et al. 2003; Press et al. 2008; Zeller et al. 2014) or γ-band responses to a visuotactile detection task in relation to the illusion (Kanayama et al. 2007, 2009) and are not directly comparable to the present ECoG results.

Using ECoG is ideal for examining the precise time course of activity around the illusion onset and in relation to the applied tactile stimulation, which has remained unclear due to the limited temporal resolution of previously used hemodynamic markers of brain activity (Ehrsson et al. 2004, 2005; Tsakiris et al. 2007; Brozzoli et al. 2012; Gentile et al. 2013). Visual inspection of the time courses of high-γ activity in the IPS (Fig. 2*C*) and PMC electrodes (Fig. 2*D*) shows that the button press that indicates the illusion onset in the synchronous condition is associated with an increase in high-γ activity compared with the asynchronous and rotated control conditions, and this elevated level of activity is sustained throughout the stimulation block. As expected, this high-γ activity increase around the illusion onset was only observed in the multisensory PMC and IPS, and not in the unimodal hand-SI cortex (Fig. 2*B*). A post hoc test showed that the high-γ activity in the hand-SI cortex, which was localized using electrical brain stimulation in this participant (P1), was not significantly modulated by the illusion experience (Fig. 2*E*), which is in contrast to the hypothesis that SI has a key role in the subjective ownership experience in the RHI (Tsakiris et al. 2007; Shokur et al. 2013). In the PMC, high-γ activity specific to the synchronous illusion condition was observed both during and in-between individual touches (Fig. 2*G*), while the illusion-specific high-γ activity in the IPS appeared to be more time-locked to the tactile stimulation (Fig. 2*E*,*F*). Descriptive post hoc tests among all significant IPS and PMC electrodes showed that high-γ activity was significantly increased both during as well as in-between individual touches in both areas in the synchronous condition (Fig. 3*A*,*B*). This finding demonstrates that the RHI is reflected in a continuously elevated level of neuronal activity in the IPS and PMC that is sustained even in-between individual touches, which is an issue that previous fMRI and PET studies have not been able to address due to limited temporal resolution (Ehrsson et al. 2004; Tsakiris et al. 2007; Guterstam et al. 2013; Limanowski and Blankenburg 2016). To examine whether illusion activity in the IPS is modulated by the delivered tactile stimulation differently than the PMC, we estimated the illusion- and touch-specific high-γ response using a “sliding window” approach because peripheral tactile stimulation takes approximately 200 ms to activate multisensory neurons in the IPS (Duhamel et al. 1998). The results of this post hoc analysis showed that the illusion-specific high-γ activity in the IPS as compared with the PMC was significantly more related to the tactile stimulation for forward-shifts the “touch data window” by 130–300 ms (Fig. 3*C*), which is consistent with the known temporal activation properties of bimodal IPS neurons (Duhamel et al. 1998). We propose that illusion-specific activity in the IPS is more related to changes in the processing of self-specific somatosensory signals originating from the “owned” rubber hand, while premotor activity reflects the continuous feeling of ownership of it. This interpretation is compatible with previous studies showing that the magnitude of the BOLD response correlates with subjective ownership ratings in the premotor cortex (Ehrsson et al. 2004; Brozzoli et al. 2012; Guterstam, Björnsdotter, Bergouignan, et al. 2015; Guterstam, Björnsdotter, Gentile, et al. 2015) and with proprioceptive drift magnitudes in the posterior parietal cortex (Brozzoli et al. 2012). Thus, these 2 areas may serve distinct functional roles in the process of self-attributing limbs, in which the PMC is the hierarchically highest level of the multisensory body representation targeted.

Our connectivity analysis revealed that activity in the hand section of SI significantly predicted activity in the posterior IPS approximately 200 ms later in an illusion-specific manner (Fig. 5*A*). Interestingly, the same IPS electrode (#50 in P1; Fig. S1) had significantly increased mean high-γ activity during the illusion in a separate independent analysis (Fig. 2*A*,*C*,*F*), while the level of high-γ activity in SI did not significantly differ across the illusion and control conditions (Fig. 2*A*,*B*,*E*). The delay of 200 ms for SI signals to reach the IPS is aligned with previous estimations of cortical processing speed of tactile stimuli (Duhamel et al. 1998). Intriguingly, the identified cross-correlation difference was driven by a strong positive correlation between the SI and IPS signals before and an equally strong negative correlation after the illusion onset (Fig. 5*C*), which was not observed in the asynchronous control condition (Fig. 5*D*), and that was primarily driven by the α-, β-, and high-γ frequency components of the signals (Fig. S4). Thus, SI influences activity in the IPS before as well as after the illusion onset, but the nature of the influence dramatically changes once the rubber hand is represented as “self.” We speculate that this finding reflects a change in how tactile signals are integrated in a multisensory hand representation in the IPS during the initial period leading up to the RHI compared with the period when ownership is established. During the initial phase of visuotactile stimulation, tactile signals from the real hand are in conflict with visual information from the rubber hand being touched. Thus, they constitute “prediction error signals” (Friston 2009; Apps and Tsakiris 2014) that contribute to updating the body-centered spatial reference frames within the hand representation in IPS, which is reflected in the strong, positive SI-to-IPS correlation in the *SynchPRE* condition in our data. Then, the initial mismatch between vision and touch is likely reconciled by remapping the corresponding spatial reference frames from the real hand to be centered on the rubber hand, which results in ownership of the rubber hand and a change in how tactile signals are integrated in the IPS (Graziano 2000; Botvinick 2004; Ehrsson et al. 2004; Makin et al. 2008; Brozzoli et al. 2012; Ehrsson 2012). Once ownership is established, the tactile signals convey less “surprise” and no longer contribute to updating the hand representation in IPS; instead, they probably reinforce and maintain the new hand representation (Friston 2009; Apps and Tsakiris 2014), which could be reflected in the transition from positive correlation in *SynchPRE* to negative correlation in *SynchPOST* between SI and subsequent IPS activity in our data. However, it should be noted that we were only able to explore the SI–IPS connectivity dynamics in one single subject (P1) due to the limitations in electrode coverage in the majority of the participants (P2–P5), and these findings should therefore be considered preliminary and interpreted with caution. Future experiments that replicate these results in additional participants are needed to support this mechanistic hypothesis for how early sensory signals are integrated in higher order cortical areas to shape the feeling of limb ownership.

In summary, this is the first intracranial electrophysiological study of the neural mechanisms that underlie the feeling of limb ownership. The results show that activity in neuronal populations in the premotor and intraparietal cortices reflect the feeling of ownership of a seen limb and provide a direct link between ownership-related BOLD responses and electrical activity that is recorded at the cortical surface. Furthermore, intraparietal activity was more strongly modulated by tactile stimulation than the premotor cortex, which suggests different functional roles of these 2 key areas in the process of attributing ownership to an artificial limb. Taken together, these findings shed new light on the dynamic neural mechanisms that support a fundamental aspect of human self-consciousness: the perception of one’s body being part of the self.

## Supplementary Material

Supplementary Data
