# Supplementary material for: Direct Electrophysiological Correlates of Body Ownership in Human Cerebral Cortex
Source: Cereb Cortex. 2018 Nov 14;29(3):1328–41. doi: 10.1093/cercor/bhy285 (PMC6373693; doi:10.1093/cercor/bhy285)
Supplement: Supplementary Data [file bhy285supplement_1.zip › bhy285_Guterstam_Supplementary_Results.docx]

| **Anatomical region** | *SynchPOST* vs *AsynchPOST* | | *SynchPOST* vs *RotatedPOST* | | **Participant** | **Electrode #** |
| --- | --- | --- | --- | --- | --- | --- |
|  | ***t*** | **p** | ***t*** | **p** |  |  |
| *Parietal lobe* |  |  |  |  |  |  |
| L. IPS, posterior section* | 5.75 | <0.001 | 6.97 | <0.001 | P1 | 50 |
| L. IPS, anterior section* | 4.41 | <0.001 | 3.51 | <0.001 | P1 | 53 |
| L. IPS* | 3.66 | <0.001 | 2.10 | 0.038** | P5 | 8 |
|  |  |  |  |  |  |  |
| *Frontal lobe* |  |  |  |  |  |  |
| R. precentral sulcus, superior end (dPMC)* | 3.19 | 0.002 | 3.12 | 0.002 | P2 | 42 |
| R. medial frontal gyrus | 4.35 | <0.001 | 4.63 | <0.001 | P2 | 4 |
| L. superior frontal sulcus, caudal portion (dPMC)* | 5.09 | <0.001 | 4.89 | <0.001 | P3 | 8 |
| L. medial frontal gyrus | 3.47 | <0.001 | 3.06 | 0.003 | P3 | 6 |
| L. medial frontal gyrus | 4.71 | <0.001 | 4.52 | <0.001 | P3 | 7 |
| L. medial frontal gyrus | 5.05 | <0.001 | 5.15 | <0.001 | P3 | 15 |
| L. medial frontal gyrus, rostral portion | 3.39 | 0.001 | 5.20 | <0.001 | P3 | 10 |
| L. medial frontal sulcus, rostral portion | 5.26 | <0.001 | 3.69 | <0.001 | P3 | 18 |
| L. precentral sulcus, inferior end (vPMC)* | 4.10 | <0.001 | 3.39 | 0.001 | P4 | 21 |
| L. superior frontal gyrus | 3.84 | <0.001 | 3.55 | <0.001 | P4 | 59 |

**Table S1**. **Areas with increased neuronal activity during the RHI (related to Figure 2).** All electrodes showing high-γ activity that was specific to the RHI, which was defined as a significantly increased high-γ power in the *SynchPOST* condition compared to both the *AsynchPOST* and *RotatedPOST* conditions. We controlled for multiple comparisons using the FDR correction. Within the PMC and IPS ROIs, we used the ROI electrodes as the search space; outside of the ROIs, we applied FDR-correction across all electrodes. Notably, five of the six significant ROI electrodes (*) also survived FDR-correction across all electrodes, with the exception of the *SynchPOST* versus *RotatedPOST* contrast for electrode #8 in P5 (**). The weaker high-γ activity difference in this single contrast may be because P5 actually experienced a weak illusion also in the rotated control condition, in which he gave an average illusion statement rating of 0 compared to 2.3 and -3 in the synchronous and asynchronous conditions. Furthermore, the fMRI results obtained in P5 showed in general much stronger illusion-related BOLD activations in the right hemisphere that was contralateral to the electrode grid (Table S2 and Figure 4C), which might also explain the weaker ECoG results in this participant. See Figure S1 for the anatomical location of the respective electrode numbers. The p values displayed are the actual (uncorrected) ones (all surviving p<0.05 FDR-correction).

| **Anatomical region** | **MNI** | **Peak *t*** | **Cluster size** | **p**_uncorrected_ | **p**_corrected_ |
| --- | --- | --- | --- | --- | --- |
| **Participant 3:** |  |  |  |  |  |
| *Parietal lobe* |  |  |  |  |  |
| R. postcentral sulcus | 16 -50 74 | 3.44 | 62 | <0.001 |  |
| R. IPS | 52 -34 56 | 2.54 | 8 | 0.006 |  |
| L. supramarginal gyrus | -62 -34 30 | 3.10 | 56 | 0.001 |  |
|  |  |  |  |  |  |
| *Frontal lobe* |  |  |  |  |  |
| R. precentral sulcus, superior end (dPMC) | 28 0 66 | 3.08 | 15 | 0.001 |  |
| L. superior frontal sulcus, caudal portion (dPMC) | -28 2 64 | 3.54 | 105 | <0.001 | 0.045^a^ |
| L. inferior frontal sulcus, rostral portion | -42 44 -6 | 2.69 | 21 | 0.004 |  |
|  |  |  |  |  |  |
| *Cerebellum* |  |  |  |  |  |
| L. cerebellum (lobe VI) | -24 -74 -24 | 2.49 | 3 | 0.007 |  |
|  |  |  |  |  |  |
| **Participant 5:** |  |  |  |  |  |
| *Parietal lobe* |  |  |  |  |  |
| R. IPS | 22 -62 60 | 3.85 | 4345 | <0.001 |  |
| R. supramarginal gyrus | 62 -20 22 | 5.56 | 4345 | <0.001 | 0.003^b^ |
| L. IPS | -40 -42 66 | 3.58 | 230 | <0.001 | 0.037^a^ |
|  |  |  |  |  |  |
| *Frontal lobe* |  |  |  |  |  |
| R. precentral sulcus, inferior end (vPMC) | 54 12 38 | 4.52 | 2343 | <0.001 | 0.041^b^ |
| R. precentral sulcus, superior end (dPMC) | 24 8 68 | 4.59 | 1301 | <0.001 | 0.041^b^ |
| R. orbitofrontal gyrus | 14 38 -18 | 5.26 | 914 | <0.001 | 0.005^b^ |
| L. precentral sulcus, superior end (dPMC) | -30 -10 62 | 3.24 | 213 | 0.001 |  |
|  |  |  |  |  |  |
| *Occipital lobe* |  |  |  |  |  |
| L. lateral occipital cortex, cluster extending into the inferior temporal gyrus | -46 -76 -6 | 4.40 | 2158 | <0.001 | 0.041^b^ |

**Table S2**. **Areas with increased fMRI-BOLD responses during the RHI (related to Figure 4).** All brain regions (peaks) in P3 and P5 surviving the statistical threshold of p<0.01 (uncorrected) in purely descriptive single subject analyses. The anatomical location of each peak was determined based on the location within the individual (non-normalized) brains. The MNI coordinates (x, y and z) for each peak were then obtained by transforming the native coordinates to those of MNI space using SPM8.  ^a^small-volume corrected using 16 mm spheres that were centered on activation peaks from (4). ^b^FDR-correction using the entire brain as a search space.

| **Participant** | **Electrode #** | **Anatomical location** | ***t*-value** | **p value** |
| --- | --- | --- | --- | --- |
| P1 | 48 | PMC | 3.11 | 0.0025 |
|  | 51 | IPS | 3.26 | 0.0016 |
|  | 52 | IPS | 3.16 | 0.0021 |
|  | 53 | IPS | 4.23 | <0.001 |
|  |  |  |  |  |
| P2 | - | - | - | - |
|  |  |  |  |  |
| P3 | 8 | PMC | 3.82 | <0.001 |
|  |  |  |  |  |
| P4 | 21 | PMC | 3.82 | <0.001 |
|  |  |  |  |  |
| P5 | 15 | IPS | 3.27 | 0.0015 |

**Table S3**. **High-γ activity after versus before illusion onset in the PMC and IPS.** Because it took about 18s for the illusion to start (group mean±SEM: 17.8±11.0s), we compared the high-γ activity recorded after the onset of the illusion with that recorded before it commenced. To eliminate nonspecific time effects, we used the data from the asynchronous as a control. Thus, we compared the *SynchPOST* - *AsynchPOST* difference with the *SynchPRE* - *AsynchPRE* difference in high-γ activity, yielding the following contrast: (*SynchPOST*>*AsynchPOST*)>(*SynchPRE*>*AsynchPRE*). Four out of five participants showed an increase in high-γ activity after the illusion onset that was specific to the synchronous condition. The spatial location of these activations closely matched the activity revealed in the main analysis that compared high-γ power in the *SynchPOST* condition compared to both the *AsynchPOST* and *RotatedPOST* conditions (Figure 2A and Table S1); either overlapping in the same electrodes (P1, P3 and P4) or in directly neighboring electrodes (P5). These results strengthen the conclusion that the feeling of owning an artificial limb is reflected in high-γ activity in the PMC and IPS. We speculate that the lack of any significant electrode in P2 was related to the extremely short illusion onset time in this participant (mean±SEM: 4.2±0.6s). Given that it probably takes a couple of seconds from the perceptual onset of the illusion until the participant decides to press the button, it is likely that a major portion of data points labelled as “pre-illusion onset” in this subject actually reflected “post-illusion onset” data points, which decreases the meaningfulness of a pre-versus-post-illusion onset analysis. The p values are the actual (uncorrected) ones (all survived p<0.05 FDR-correction across all grid electrodes).

**
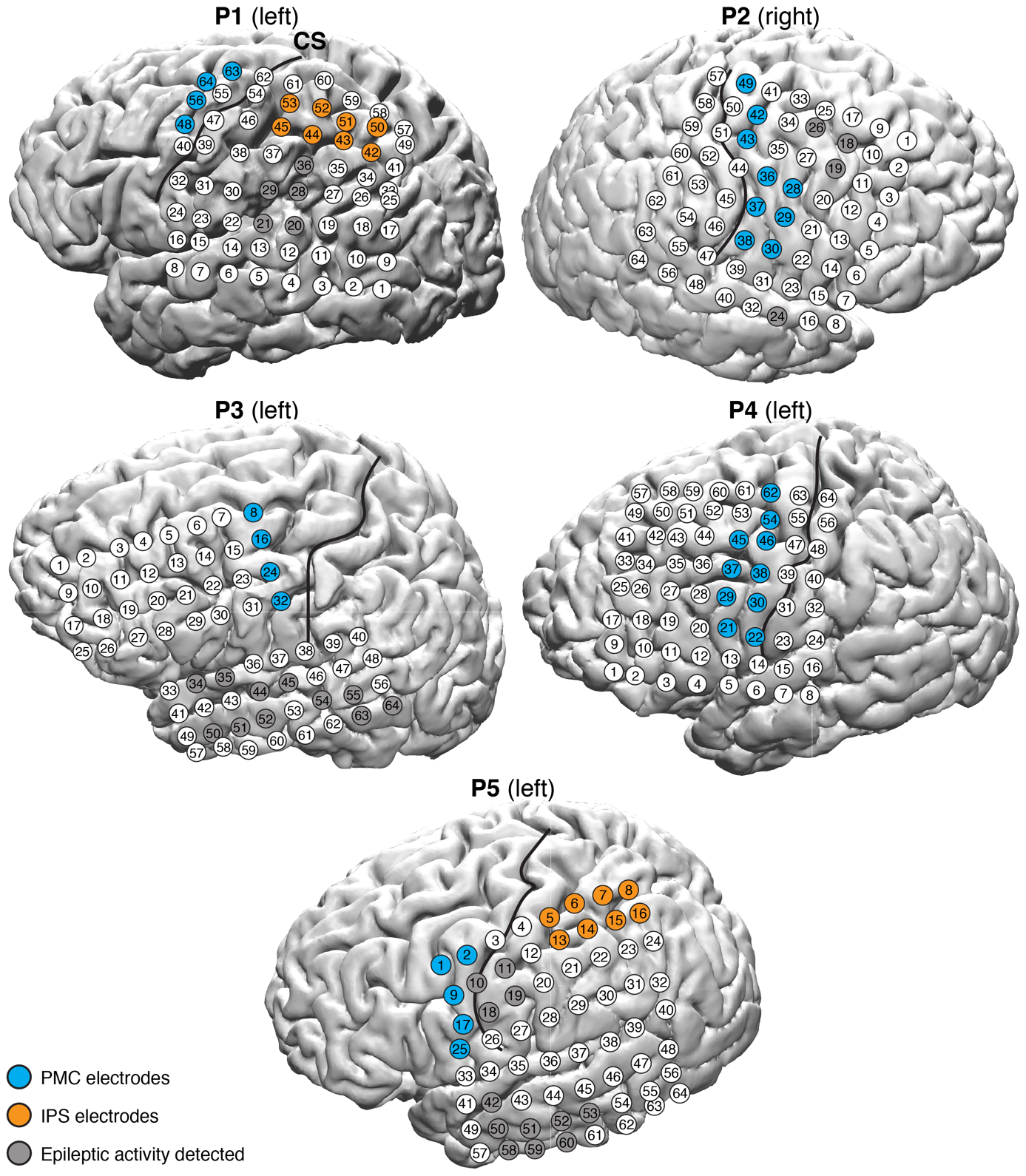
**

**Figure S1.** **Regions of interest (ROIs)**. The electrodes belonging to the ROIs, which consisted of the premotor cortex (PMC) and the intraparietal sulcus (IPS), were selected based on their anatomical locations. CS=central sulcus.


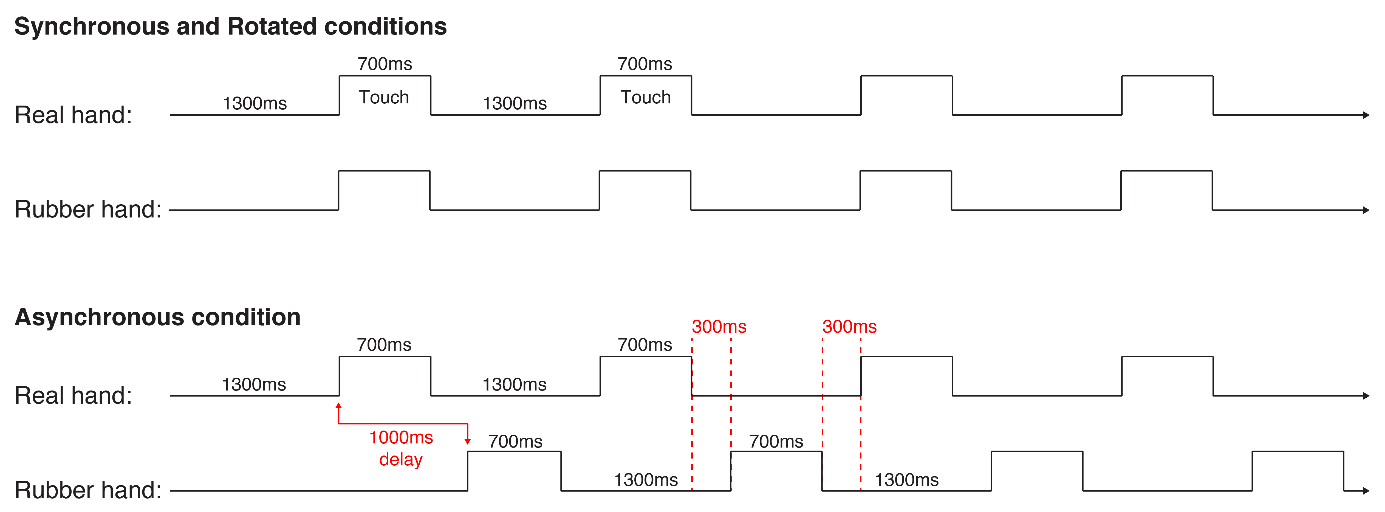


**Figure S2. Experimental conditions and visuo-tactile timing.** The timing of the touches that were delivered to the real hand and the rubber hand in the three experimental conditions are shown. The touches were delivered in synchrony in the synchronous and rotated conditions. In the asynchronous condition, the tactile stimulation was delayed by 1000 ms relative to the touches that were delivered to the rubber hand.

**
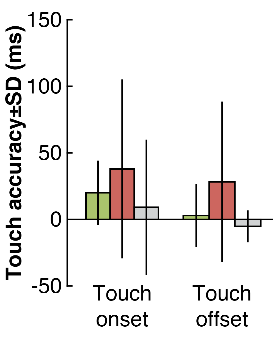
**

**Figure S3.** **Experimenter accuracy**. The manually delivered strokes were applied with a high degree of synchrony (<50 ms), and the timing, in terms of real versus rubber hand touch onset and offset, did not significantly differ across experimental conditions (p>0.05). The asynchronous touches are adjusted by 1000 ms for display purposes. Green: synchronous condition; Red: asynchronous condition; Grey: Rotated condition.

**
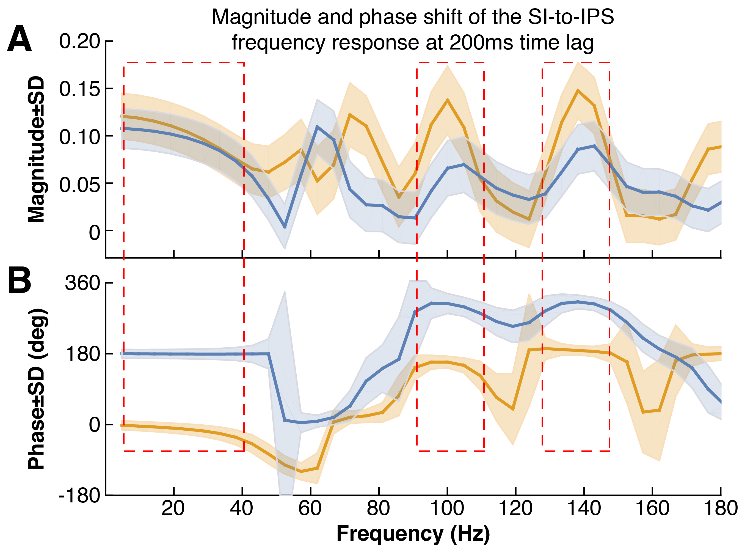
**

**Figure S4. Frequency response difference (related to Figure 5C).** The *SynchPRE* versus *SynchPOST* correlation difference that was observed at 200 ms was primarily driven by one lower and two higher frequency band spectra, as revealed by a Bode plot analysis. Blue=*SynchPRE* condition; Yellow=*SynchPOST* condition.


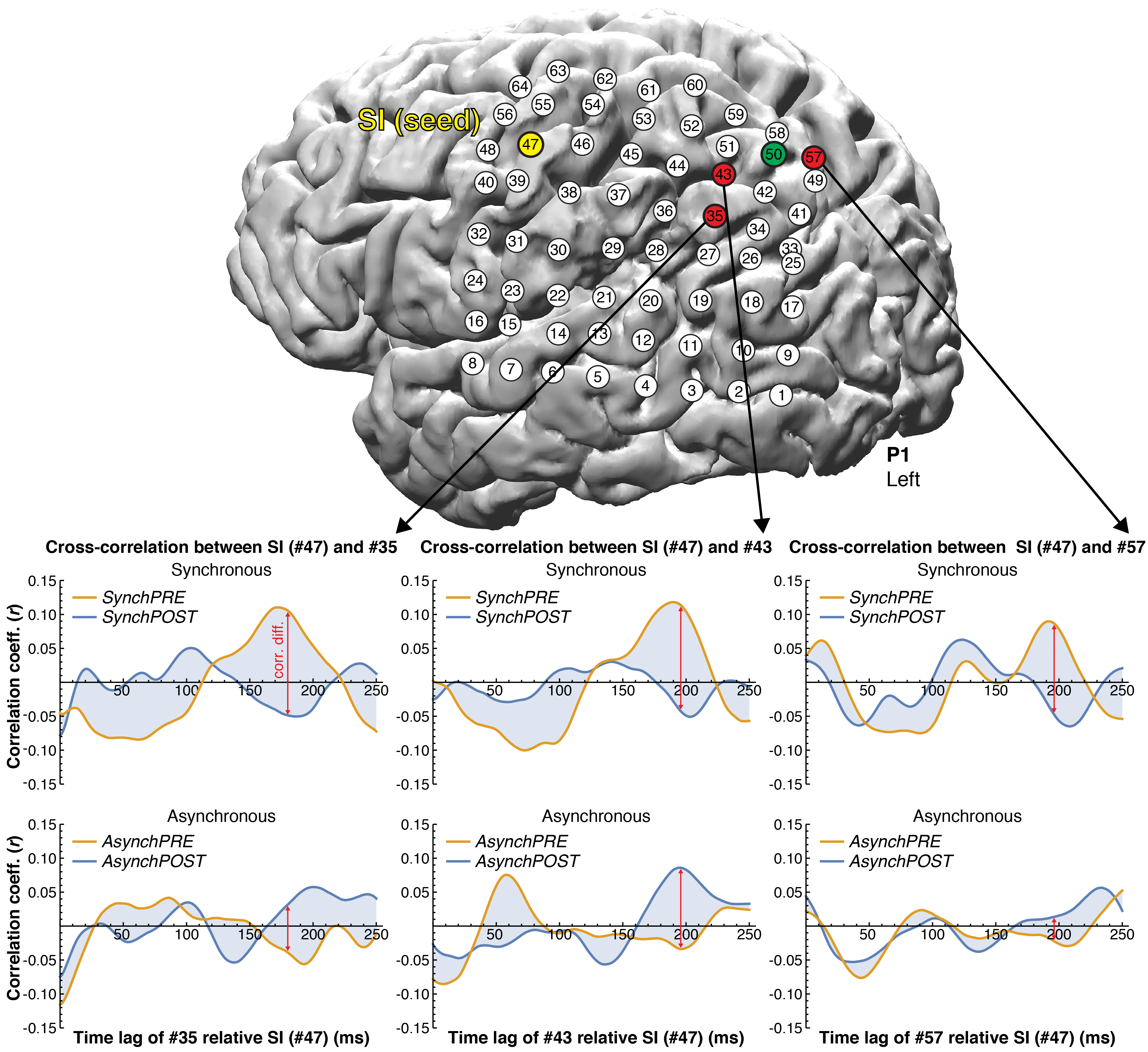
**Figure S5. Ownership-specific connectivity from the SI to the posterior parietal cortex.** As can be seen in Figure 5A, the effective connectivity analysis revealed large *SynchPOST* vs. *SynchPRE* cross-correlation differences around 200ms not only in the peak electrode #50 (located in the IPS; colored green) but also in three adjacent electrodes in the posterior parietal cortex (#57 in the superior parietal lobule, #43 in the IPS and #35 in the supramarginal gyrus; colored red). Even though the electrode #50 was of key interest because it also showed illusion-specific high-γ activity in an independent analysis (Figure 2), exploring the factors driving the *SynchPOST* vs. *SynchPRE* cross-correlation differences in the surrounding electrodes is valuable to the interpretation of the illusion-induced reversal of the correlation sign observed in #50. We hypothesized that the switch from a positive SI-IPS cross-correlation before illusion onset (*SynchPRE*) to a negative one after illusion onset (*SynchPOST*) at around 200ms time lag reflects a meaningful property of the underlying neural signal processing the parietal lobe (rather than being a ‘statistical anomaly’) and should therefore be observable also in the adjacent electrodes. In accordance with our prediction, all three electrodes (#57, #43 and #35) showed the similar reversal of the sign of the correlation as was observed in the peak electrode #50 (see upper panels). Furthermore, analyzing the corresponding periods in the asynchronous control condition revealed substantially weaker cross-correlation differences and a reversed relationship between the correlation signs during the periods before (*AsynchPRE*: slightly negative correlation) and after button press (*AsynchPOST*: slightly positive correlation) at around 200ms, which was consistent across all three electrodes (see lower panels). Together, these results are consistent of the interpretation that the SI-IPS cross-correlation finding in electrode #50 reflects of a meaningful property of the underlying neural signal processing. A potential, admittedly speculative, underlying neural mechanism is discussed in the main text.
