# Supplementary material for: Direct Electrophysiological Correlates of Body Ownership in Human Cerebral Cortex
Source: Cereb Cortex. 2018 Nov 14;29(3):1328–41. doi: 10.1093/cercor/bhy285 (PMC6373693; doi:10.1093/cercor/bhy285)
Supplement: Supplementary Data [file bhy285supplement_1.zip › bhy285_Guterstam_Supplementary_Methods.docx]

**Supplementary Materials and Methods**

**Functional MRI data acquisition.** Functional imaging data were acquired prior to neurological surgery with a Philips 3T Achieva at the Integrated Brain Imaging Center (University of Washington, Seattle, WA). All images were acquired using an 8-channel SENSE head coil. Gradient-echo T2*-weighted echo-planar images with the blood-oxygen dependent (BOLD) contrast were used as an index of brain activity (Logothetis et al. 2001). Functional image volumes were composed of 40 continuous near-axial slices with a thickness of 3 mm (with a 1 mm interslice gap), which ensured that the entire brain was within the FOV (80×80 matrix, 3.0 mm×3.0 mm in-plane resolution, TE=32 ms). One complete volume was collected every 2.50 s (TR=2500 ms). In total, 516 functional volumes were collected for each participant and were divided into two sessions. The first three volumes were discarded to account for non-steady-state magnetization. To facilitate the anatomical localization of statistically significant activations, a high-resolution structural image was acquired for each participant at the end of the experiment (3D MPRAGE sequence, voxel size=1 mm×1 mm×1 mm, FOV=256 mm×256 mm, 170 slices, TR=6.5 ms, TE=3 ms, flip angle=8**°**).

**fMRI – Experimental conditions and design.** During scanning, the participants laid in a supine position on the MRI bed with their head tilted at approximately 30**°** to allow for a direct view of an MR-compatible plastic table (42×35 cm), with an adjustable slope, which was mounted on the bed above the subject’s waist. The participants’ hand was placed on the side of the table in a fully extended posture. Great care was taken to ensure that the participant could relax the hand and that it was placed in a comfortable position. A white cloth hid the participant’s hand from view (the general procedures were identical to those in the fMRI experiment in (Guterstam et al. 2013)).

The experimental conditions in the fMRI experiment were identical to those in the ECoG experiment, specifically: *synchronous* or *asynchronous* stimulation or synchronous stimulation using a *rotated* rubber hand. Each condition was repeated eight times in a randomized order over two 10.5-min-long sessions, and the duration of each epoch was 35 s. To ensure that the participants remained alert, each session featured two catch trials in response to which the participants were instructed to press a button using their unstimulated hand. In the ‘visual’ catch trial, the probe that touched the rubber hand suddenly stopped on the middle of rubber hand’s finger for one single touch, while the touch on the real hand’s finger continued along the full length of the finger as usual. In the ‘tactile’ catch trial, the touch on the real hand suddenly stopped, while the touch on the rubber hand remained full length. The results showed that both participants in the fMRI experiment responded accurately to all of the catch trials, which demonstrated that they remained alert and attentive to the visual and tactile stimuli.

The experimenter (AG) listened to a sound track that provided the appropriate audio cues for delivering the visual and tactile stimuli and information on the forthcoming experimental condition. The timing and delivery of the touches in the fMRI and ECoG experiments were identical.

**fMRI analysis.** fMRI data were analyzed with SPM8. Following standard preprocessing without normalizing, the functional images were spatially smoothed with an 8-mm full width at half maximum isotropic Gaussian kernel. Using a single-subject analysis approach, we defined separate regressors for the *synchronous*, *asynchronous* and *rotated* epochs. Two regressors of no interest were created to model the visual and tactile catch trials. Each condition was modeled with a boxcar function and convolved with the standard SPM8 hemodynamic response function. Linear contrasts were defined in the general linear model, and the results from this analysis were provided as contrast images.

To identify the brain regions in which BOLD responses were specifically related to the RHI, we contrasted *synchronous* versus *asynchronous* (p<0.01, uncorrected) and used the *synchronous* versus *rotated* contrast (p<0.05, uncorrected) as an inclusive mask, consistent with the analysis approach described in (Guterstam et al. 2013). Importantly, the areas that were identified using this approach had greater activation in the illusion condition compared to either of the two control conditions and, thus, had an activation profile that obeyed the known spatial and temporal rules of the RHI (Botvinick and Cohen 1998; Ehrsson et al. 2004). For visualization purposes, an activation map that contained all significant voxels was overlaid onto a 3-D reconstruction of the participants’ individual brains using MRIcron (University of South Carolina, SC, USA) (Figure 4A-B). For statistical inference in the areas where we had *a priori* defined hypotheses (the PMC and IPS), we used a small-volume correction that consisted family-wise error rate correction within spheres of a 16 mm radius that were centered around the peaks from a previously published landmark study (Ehrsson et al. 2004). For peaks outside our ROIs, we applied FDR-correction using the entire brain as search space.

**Supplementary References**

Botvinick M, Cohen J. 1998. Rubber hands “feel” touch that eyes see. Nature. 391:756.

Ehrsson HH, Spence C, Passingham RE. 2004. That’s my hand! Activity in premotor cortex reflects feeling of ownership of a limb. Science. 305:875–877.

Guterstam A, Gentile G, Ehrsson HH. 2013. The Invisible Hand Illusion: Multisensory Integration Leads to the Embodiment of a Discrete Volume of Empty Space. J Cogn Neurosci. 25:1078–1099.

Logothetis NK, Pauls J, Augath M, Trinath T, Oeltermann A. 2001. Neurophysiological investigation of the basis of the fMRI signal. Nature. 412:150–157.
